# Cadmium decreases human gingival fibroblast viability and induces pro-inflammatory response associated with Akt and MAPK pathway activation

**DOI:** 10.3389/ftox.2025.1583865

**Published:** 2025-07-23

**Authors:** Tipparat Parakaw, Sirada Srihirun, Nathawut Sibmooh, Nisarat Ruangsawasdi, Phisit Khemawoot, Pornpun Vivithanaporn

**Affiliations:** ^1^ Department of Pharmacology, Faculty of Dentistry, Mahidol University, Bangkok, Thailand; ^2^ Chakri Naruebodindra Medical Institute, Faculty of Medicine Ramathibodi Hospital, Mahidol University, Samut Prakan, Thailand

**Keywords:** cadmium, fibroblasts, cyclooxygenase, interleukin-6, interleukin-8

## Abstract

Smoking and particulate matter 2.5 (PM2.5) expose millions to cadmium (Cd), a toxic heavy metal linked to pro-inflammatory responses, oxidative stress, and disease pathogenesis. In the oral cavity, chronic Cd exposure contributes to the progression of periodontal diseases and oral cancers. However, the direct effect of Cd on oral tissues and the underlying mechanisms remains unclear. This study explored the impact of environmentally relevant concentrations of Cd on human gingival fibroblasts (HGFs) by evaluating cell viability, pro-inflammatory cytokine secretion (IL-6 and IL-8), COX-2 expression, and the activation of key signaling pathways: Akt, ERK1/2, and JNK. Cd exposure significantly reduced HGF viability, elevated IL-6 and IL-8 secretion, and upregulated COX-2 expression. These effects were attenuated by inhibitors targeting Akt, ERK1/2, and JNK pathways. By integrating cytokine profiling, COX-2 expression, and inhibitor-based pathway analysis, our study provides mechanistic insights into how low-level Cd exposure triggers early inflammatory responses in gingival fibroblasts. Our findings reveal that Cd exerts pro-inflammatory and cytotoxic effects on HGFs, which may play a role as one of the factors in the pathogenesis of smoking-related oral diseases. Targeting Akt, ERK1/2, and JNK signaling pathways could offer therapeutic strategies to attenuate Cd-induced oral pro-inflammatory responses and tissue damage.

## Graphical Abstract

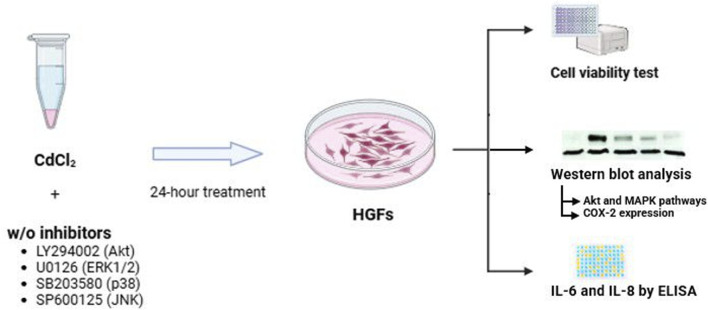

## Introduction

Smoking increases cadmium (Cd) levels, a toxic metal found in tobacco smoke at approximately 0.08 – 5.4 μg/g tobacco (Fresquez et al., 2013; Dinh et al., 2021; Richard et al., 2010). Salivary Cd is significantly higher in waterpipe and long-term smokers, reaching up to 352.72 μM which was also comparable to levels in plasma (Khabour et al., 2018; Bertuzzo, 2003). Cd levels in saliva and urine are proposed as indicators of smoking addiction, correlating with cigarette consumption (Talio et al., 2010). Salivary Cd levels were 0.03 μM in subjects smoking 3-5 cigarettes daily and 0.08-0.11 μM in those smoking 20-40 cigarettes daily, while non-smokers had levels around 0.006 μM. Cd is a known component of particulate matter 2.5 (PM2.5), linking airborne particulate to heavy metal contamination. A study showed that Tobacco smoke increases indoor PM2.5 and Cd levels in smoker households (Ruggieri et al., 2014). Moreover, prolonged exposure to these fine particles may damage oral tissues and promote dental caries (Zhu et al., 2024).

Interleukins (ILs) are key mediators of oral inflammation. IL-6 is associated with chronic inflammation, contributing to periodontal damage, periodontitis, Sjögren's syndrome, and oral squamous cell carcinoma (OSCC) (Nibali et al., 2012; Noh et al., 2013; Gasche et al., 2011). IL-8 is elevated in the gingival tissue of periodontitis patients, and both IL-6 and IL-8 are potential biomarkers for oral and oropharyngeal squamous cell carcinoma (Sahibzada et al., 2017; St John et al., 2004; Finoti et al., 2017). Smoking increases IL-6 and IL-8 levels in cells, animals, and humans, with smokers showing significantly higher levels in saliva compared to non-smokers (Hussein et al., 2020). Cd exposure has been shown to trigger IL-6 and IL-8 expression and secretion via MAPK and NF-κB pathways in neutrophils, macrophages, epithelial, and astrocytoma cells (Hossein-Khannazer et al., 2020; Olszowski et al., 2012; Phuagkhaopong et al., 2017), highlighting its role in inflammation.

COX-2 upregulation drives inflammation. In periodontitis, its increased expression correlates with alveolar bone loss in rat models (Li et al., 2021). Cd induces COX-2 in a dose-dependent manner in human gallbladder cells and activates the p38 MAPK pathway in brain endothelial cells (Sharma et al., 2020; Seok et al., 2006). These findings highlight the underlying mechanisms of Cd on COX-2 expression and its role in inflammatory responses.

While nicotine is widely recognized for its role in gum inflammation and periodontal disease, the contribution of Cd remains less defined despite the oral cavity being a primary site of Cd exposure through tobacco smoke and PM2.5. To address this gap, we investigated how Cd induces pro-inflammatory responses in human gingival fibroblasts (HGFs), focusing on cytokine secretion and activation of MAPK pathways. Unlike earlier studies that examined cytotoxicity, pro-inflammatory responses, or signaling in isolation (Issa et al., 2008; Yang et al., 2025; Chmielowska-Bąk and Deckert, 2012; Chen et al., 2022), our work integrates these aspects using environmentally relevant Cd concentrations. This approach provides clearer insight into the pro-inflammatory effects of Cd in the oral environment and their relevance to smoking-related and environmental health risks.

## Materials and methods

### Reagents

HGF cells obtained from ATCC CRL-2014, USA were used in this study. Culture media and supplements including Dulbecco’s Modified Eagle Medium (DMEM), fetal bovine serum (FBS), penicillin, streptomycin, and L-glutamine were purchased from Sigma-Aldrich (USA), Invitrogen (USA), and Gibco (USA). Cadmium chloride (CdCl_2_), MTT reagent were purchased from Sigma-Aldrich (USA), while dimethyl sulfoxide (DMSO) was obtained from LGC (UK). Specific pathway inhibitors: LY294002 (Akt), U0126 (ERK1/2), SB203580 (p38), and SP600125 (JNK) were purchased from Calbiochem (USA), Cell Signaling Technology (USA), Tocris (UK), and Sigma-Aldrich (USA), respectively. ELISA kits for IL-6 and IL-8 were from eBioscience (USA). Primary antibodies for signaling proteins and COX-2 were obtained from Cell Signaling Technology (USA) and Clarity ECL western blot substrate kits purchased from Bio-Rad (USA) were used as chemiluminescent reagents.

### Cell culture

HGF cells at passages 3–10 were maintained in complete DMEM, with media refreshed every 2 days. Cells were incubated at 37°C in a humidified atmosphere with 5% CO_2_ until reaching 90% confluence.

A schematic overview of the experimental workflow is provided in Graphical Abstract for clarity.

### Cell viability test

HGF cells (1x10^4^ cells) were cultured in a 96-well plate for 24 h and treated with CdCl_2_ (0, 0.1, 1, 3, 6, 10, and 100 μM) for 24 h. CdCl_2_ was selected as the Cd source due to its stability and water solubility, providing a consistent release of Cd^2+^ ions which is suitable for *in vitro* toxicological studies. A 1000 mM stock solution was prepared in sterile water and stored at −20°C until use. Cell viability was assessed using the MTT assay (0.5 mg/ml for 2 h at 37°C) followed by DMSO to dissolve crystals. Absorbance was measured by a microplate reader (Biotek Instruments, CA, United States) at 570 nm (background 690 nm). DMEM without CdCl_2_ served as the control (100% viability). In addition, HGFs were co-treated with CdCl_2_ and inhibitors dissolved in DMSO (LY294002: Akt inhibitor, U0126: ERK1/2 inhibitor, SB203580: P38 inbibitor, and SP600125: JNK inhibitor) and cell viability was determined at 24 h.

### Western blot analysis

HGF cells were cultured in 60-mm dishes and treated with 1 μM CdCl_2_ to measure Akt, ERK1/2, p38, and JNK with or without their specific inhibitors. 1 µM Cd was selected as it represented the highest concentration that did not cause cell death in our experiment. Protein lysates were collected and stored at −80°C. For western blotting, 20 μg protein lysates were separated using SDS-PAGE and transferred to a nitrocellulose membrane. The transferred membranes were blotted for phospho-Akt, phospho-Erk, phospho-p38, phospho-JNK, total-Akt, total-Erk, total-p38, total-JNK, β-actin, β-tubulin, or GAPDH. Additionally, COX-2 expression was measured after 1-hour CdCl_2_ treatment, with or without inhibitors. COX-2 antibody and GAPDH were detected. Antibodies were from Cell Signaling, USA, and chemiluminescent signals were visualized using the Amersham ImageQuant 800 Fluor visualizer (Cytiva, United States), with densitometry quantified by ImageJ.

### Measurement of IL-6 and IL-8 by ELISA

HGF cells (1x10^4^ cells) seeded in 96-well plates were co-treated with 1 μM CdCl_2_ and their specific inhibitors for 24 h. The supernatants were collected and centrifuged for subsequent ELISA analysis. The ELISA procedure was performed according to the manufacturer’s instructions. Briefly, 96-well plates were coated overnight with IL-6 or IL-8 capture antibodies. After washing and blocking the plates, standards or supernatants were added for 2 h, followed by detection antibodies for 1 h. HRP-labeled antibodies were incubated for 30 min. After washing, substrate was added, and the reaction stopped with 1 M sulfuric acid. Optical density was measured at 450 nm (background at 570 nm), and cytokine levels were interpolated from standard curves.

### Statistical analysis

Statistical analyses were performed using Prism® version 10.3.1 (Prism Software Inc., United States). Data are presented as mean ± SEM. The IC50 of CdCl_2_ was determined using nonlinear regression. One-way ANOVA with Dunnett’s test was applied to evaluate cell viability (MTT assay) and IL-6 and IL-8 secretion (ELISA). For western blot analysis, one-way ANOVA with Tukey’s test was employed. A p-value of ≤ 0.05 was considered significant.

## Results

### Cd was cytotoxic to HGF cells

**FIGURE 1:**
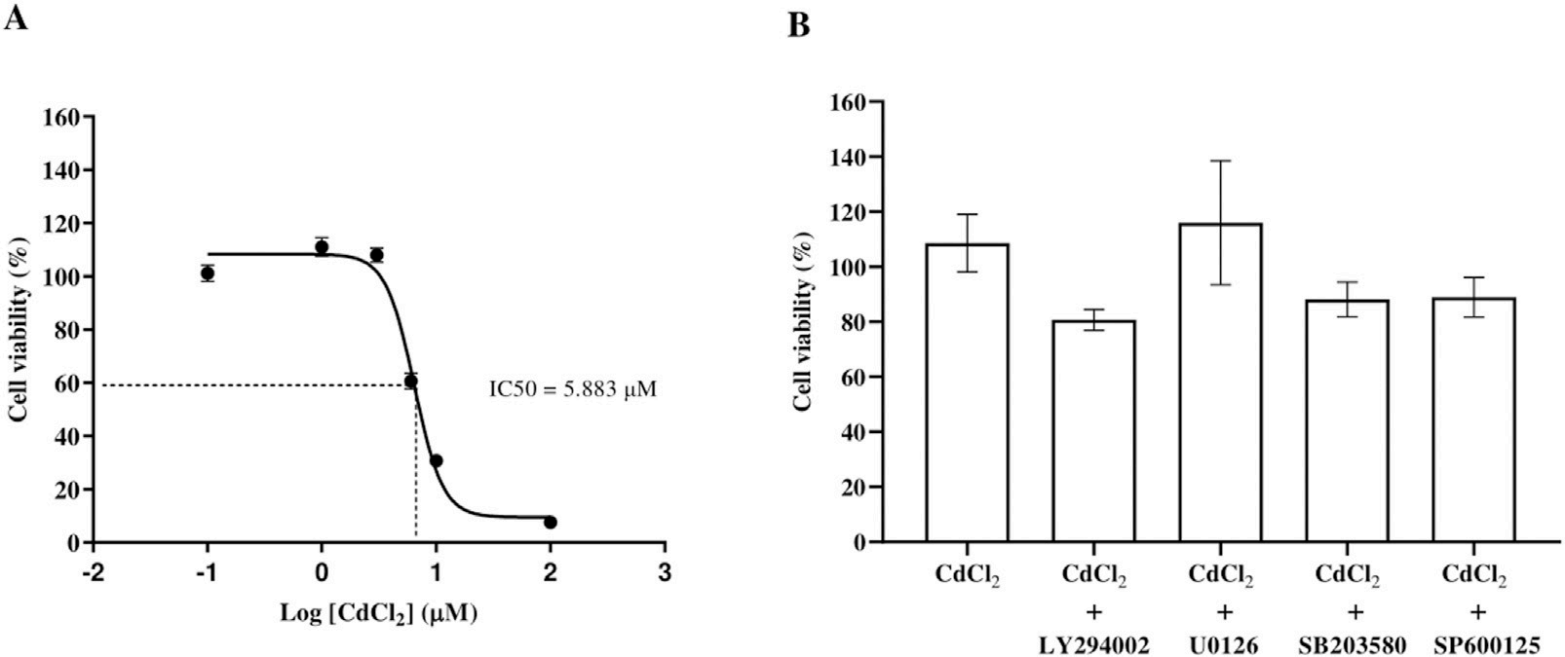
Dose-response curve of CdCl_2_-induced cytotoxicity in HGF and cell viability of HGFs following exposure to Cd and inhibitors. Cell viability was assessed after exposure to increasing concentrations of CdCl_2_ (0, 0.1, 1, 3, 6, 10, and 100 μM) for 24 h using MTT assays. The IC_50_ value, representing the concentration of CdCl_2_ required to reduce cell viability by 50%, was determined from the dose-response curve **(A)**. The viability of HGFs was also assessed after treatment with 1 µM CdCl_2_ alone or in combination with specific pathway inhibitors: 10 µM LY294002 (Akt), 25 µM U0126 (ERK1/2), 25 µM SB203580 (p38), and 25 µM SP600125 (JNK) **(B)**. HGFs cultured in control (DMEM) without treatment were the control group representing 100% cell viability. Data are shown as mean ± SEM (n = 3).

CdCl_2_ reduced HGF viability in a concentration-dependent manner, with significant cytotoxicity at ≥ 6 μM. The IC50 was 5.883 μM (Figure 1A). Since 1 μM CdCl_2_ was not cytotoxic, it was used in subsequent pro-inflammatory responses experiments. Co-treatment with inhibitors at 1 μM CdCl_2_ showed no significant effect on cell viability (Figure 1B). Although some values slightly exceeded 100%, they were not statistically significant compared to controls and likely reflect normal experimental variability.

### Cd activated Akt and MAPK pathways in HGFs

**FIGURE 2:**
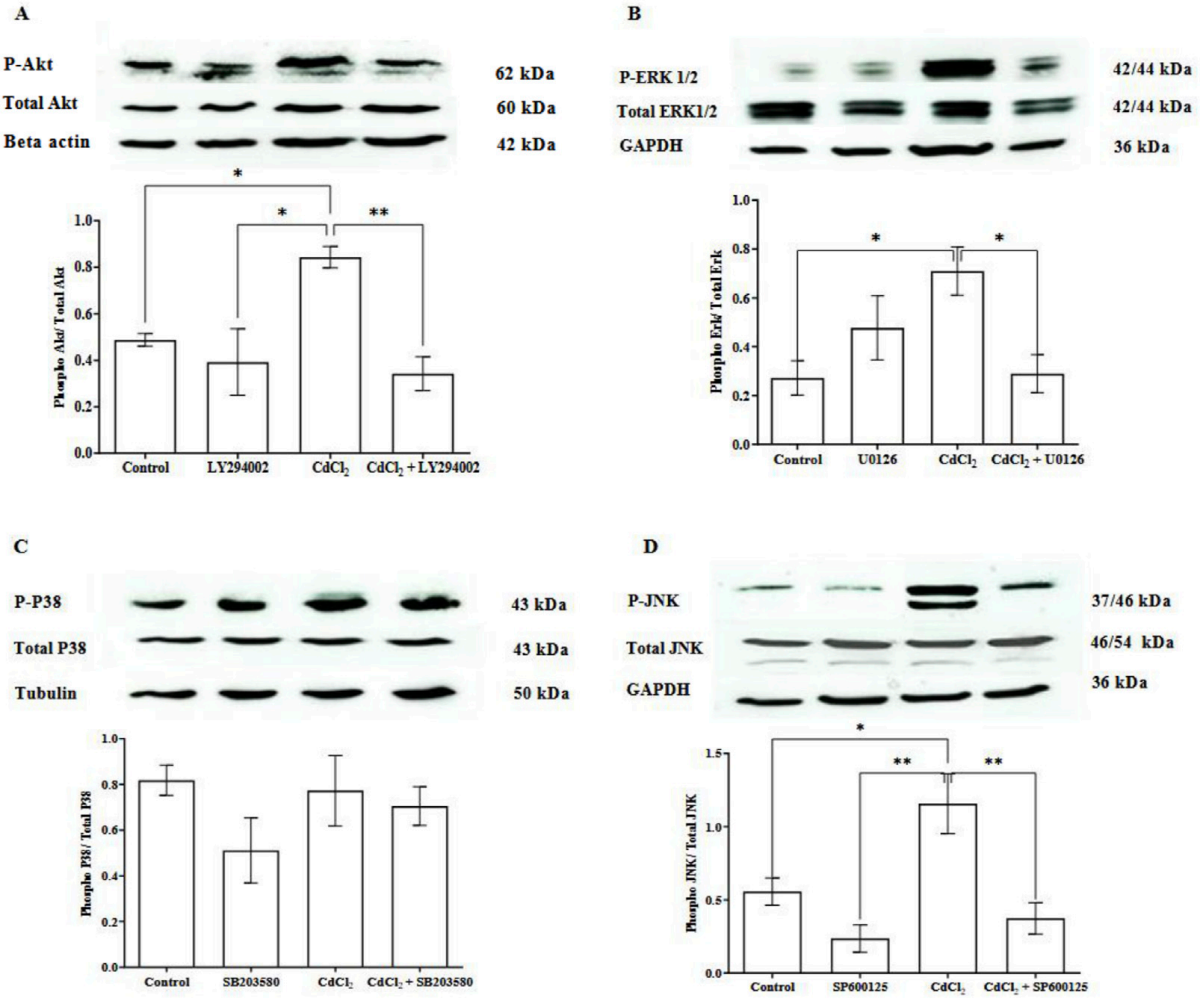
CdCl_2_-induced Akt, ERK 1/2, and JNK expression activation in cultured HGFs. HGF cells were incubated with 1 μM CdCl_2_ or co-treatment of 1 μM CdCl_2_ and each inhibitor: 10 µM LY294002 (Akt), 25 µM U0126 (ERK1/2), 25 µM SB203580 (p38), and 25 µM SP600125 (JNK). HGF cells were also treated with the inhibitor alone to determine its effect. Control refers to cells cultured in DMEM without CdCl_2_ treatment. Cells were incubated for 5 min for phosphorylated Akt measurement and 30 min for phosphorylated ERK1/2, p38, and JNK measurement. Levels of phospho-Akt, total Akt **(A)**, phospho-ERK 1/2, ERK **(B)**, phospho-p38 MAPK, p38 MAPK **(C)**, and phospho-JNK, JNK **(D)** were investigated by Western blotting. Beta actin, GAPDH, and tubulin were used as housekeeping genes based on molecular weights of proteins of interest. Data are shown as mean ± SEM (n = 4). Statistical significance was determined using One-way ANOVA followed by Tukey’s multiple comparisons test represented by *P ≤ 0.05, **P ≤ 0.01.

To study the pathways involved in Cd-induced effects on HGFs, we treated the cells with 1 μM CdCl_2_, alone or in combination with their specific pathway inhibitors. The treatment with 1 μM CdCl_2_ significantly increased the phosphorylation of Akt, ERK1/2, and JNK (Figure 2). Their specific inhibitors effectively blocked the activation of phosphorylated Akt, ERK1/2, and JNK (LY294002, U0126, SP600125, respectively). However, CdCl_2_ did not increase phosphorylated p38 levels, and treatment with SB203580 did not affect these levels.

### Cd induced IL-6 and IL-8 secretion from HGFs which were decreased by Akt and MAPK inhibitors

**FIGURE 3:**
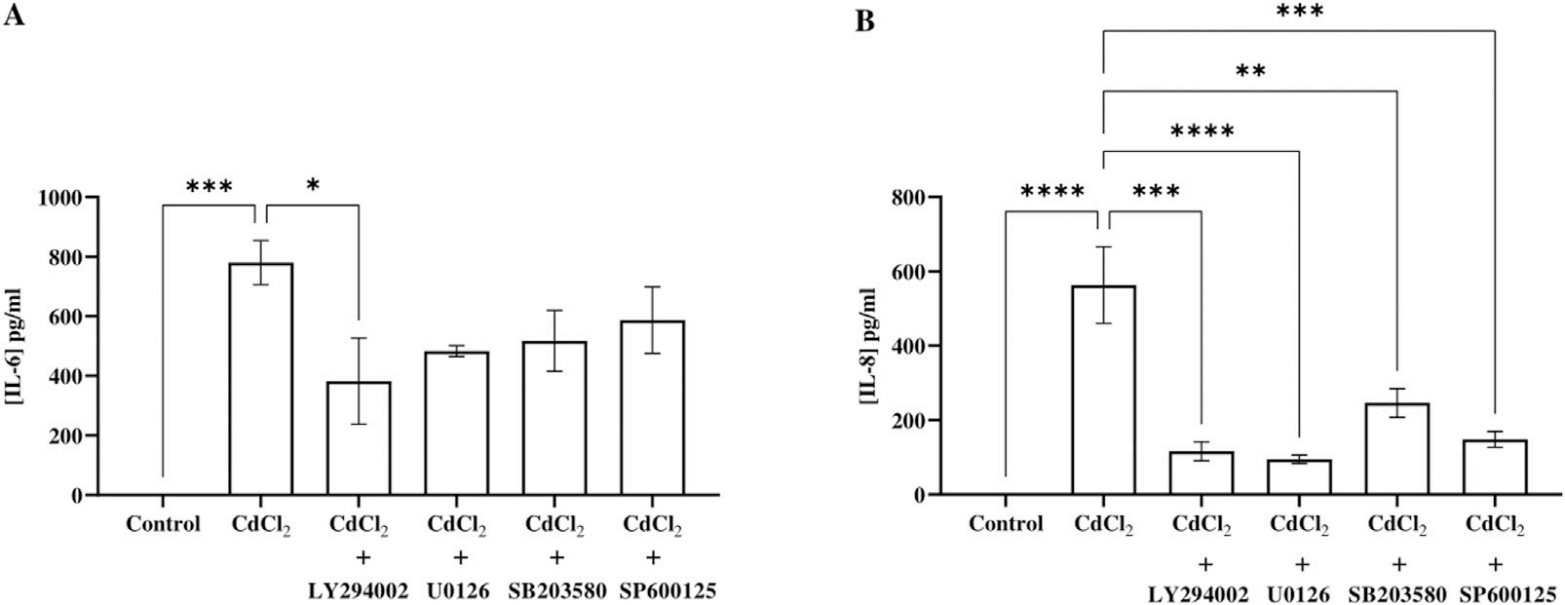
IL-6 and IL-8 secretion response to CdCl_2_ exposure and the effect of inhibitors. HGF cells were co-treated with 1 µM CdCl_2_ and specific inhibitors: 10 µM LY294002 (Akt), 25 µM U0126 (ERK1/2), and 25 µM SP600125 (JNK) prior to the measurement of IL-6 **(A)** and IL-8 levels **(B)** by ELISA. Control refers to cells cultured in DMEM without CdCl_2_ treatment. Data are shown as mean ± SEM. For all groups n = 3. Statistical significance was determined using One-way ANOVA followed by Dunnett’s multiple comparison test represented by *P ≤ 0.05, **P ≤ 0.01, ***P ≤ 0.001.

To demonstrate the pro-inflammatory effects of Cd on HGFs, we measured IL-6 and IL-8 levels following Cd exposure. Our results revealed that CdCl_2_ (1 μM) inceased IL-6 and IL-8 secretion after 24 hours of incubation (Figure 3). Based on our previous results, 1 μM CdCl_2_ did not activate p38 MAPK pathway, so we excluded p38 inhibitor in this experiment. The results showed that all tested inhibitors: 10 µM LY294002 (Akt), 25 µM U0126 (ERK1/2), and 25 µM SP600125 (JNK) decreased both IL-6 and IL-8 levels induced by CdCl_2_ in HGFs.

### Cd increased COX-2 expression in HGFs, which was inhibited by Akt and MAPK inhibitors

**FIGURE 4:**
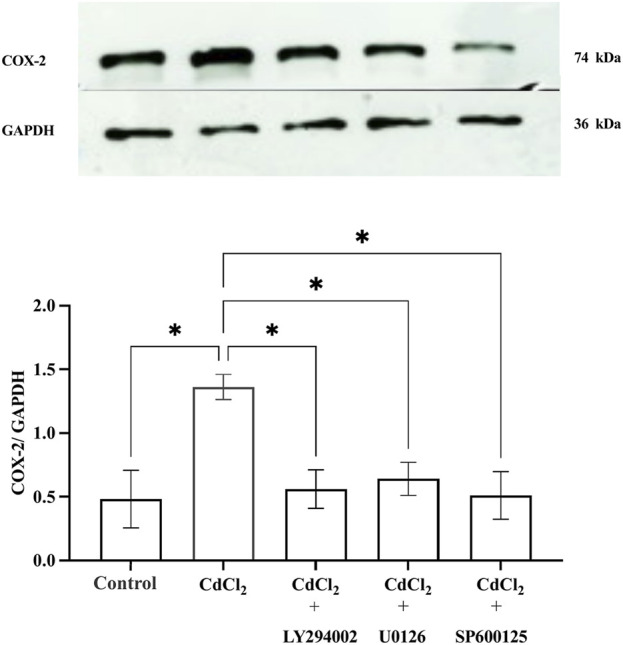
Effect of CdCl_2_ on COX-2 expression in HGFs. Western blot analysis of COX-2 expression in HGFs after 1-h incubation with 1 μM CdCl_2_, with and without specific pathway inhibitors: 10 µM LY294002 (Akt), 25 µM U0126 (ERK1/2), and 25 µM SP600125 (JNK) were determined. Control refers to cells cultured in DMEM without CdCl_2_ treatment. The upper bands represent COX-2, and the lower bands correspond to GAPDH which was used as housekeeping genes. Data are shown as mean ± SEM (n = 4). Statistical significance was determined using One-way ANOVA followed by Dunnett’s multiple comparison test represented by *P ≤ 0.05, **P ≤ 0.01.

CdCl_2_ (1 μM) treatment induced COX-2 expression in HGFs 1 h after the exposure (Figure 4). This induction of COX-2 expression was significantly attenuated when the cells were co-treated with inhibitors (10 µM LY294002 for Akt, 25 µM U0126 for ERK1/2, and 25 µM SP600125 for JNK), indicating that these pathways contribute to COX-2 upregulation in response to Cd exposure.

## Discussion

This study demonstrates that Cd exerts harmful effects on normal HGFs through the activation of specific signaling pathways. Our findings show that Cd exposure activates the phosphorylated Akt, ERK1/2, and JNK signaling pathways, which mediate subsequent cellular responses. These include a significant decrease in cell viability, the induction of pro-inflammatory cytokines IL-6 and IL-8, and an increase in COX-2 expression. Therefore, our study provides a mechanistic link between Cd exposure, pro-inflammatory responses, and the development of smoking-related oral diseases.

The cytotoxic effects of Cd on HGF cells were evident, with a dose-dependent reduction in cell viability. The cytotoxic effects of Cd in our study align with previous reports demonstrating its dose- and time-dependent toxicity in normal cell types such as human lens epithelial cells and intestinal epithelial (IEC-18) cells in a dose-dependent manner (Duizer et al., 1999; Kishimoto et al., 1994). Cd also induced cytotoxicity in normal human keratinocytes (HaCaT cells) by reducing cell viability (LC_50_ of 11 µM), causing DNA damage and apoptosis (Li et al., 2022). In addition, Cd has the potential to transform normal cells into cancer cells. Chronic exposure to 10 μM Cd turned benign prostatic hyperplasia (BPH) cells to become malignant ones by increasing their ability to form colonies, invade, and migrate (Chandrasekaran et al., 2020). Cancer cells are more susceptible to Cd's cytotoxic effects than normal cells. A study reported that 20 nM Cd reduced the viability of A549 lung cancer cells, a much lower concentration than what affected normal HGFs in our study (Kim et al., 2020). Interestingly, clinical research found Cd accumulation in the oral keratinized mucosa (OKM) at concentrations of 0.76 µM in smokers and 1.28 µM in non-smokers (Satir et al., 2024). These levels are comparable to the 1 µM Cd concentration used in our study, making it a relevant and pathologically meaningful concentration for our study. We also determined the IC_50_ value of 5.883 μM, suggesting that even low Cd exposure levels can impair cellular functions in HGFs. Reported salivary Cd levels vary considerably depending on smoking behavior and method. The results ranged from 0.08–0.11 μM in heavy cigarette smokers to over 40 μM in long-term waterpipe users, and over 300 μM in long-term heavy tobacco users (Bertuzzo, 2003; Talio et al., 2010; Khabour et al., 2018). In our study, we mainly focused on the 1 μM concentration of Cd, which falls within the higher end of reported levels and is similar to what has been found in OKM. However, we did not explore the lowest concentration (0.1 μM) in detail, even though it is also closer to salivary Cd levels in smokers reported from some reports. Future studies should include this range to better reflect typical environmental exposure. Additionally, Cd may have a comparable or even greater cytotoxic potential at lower concentrations than nicotine, a major component of tobacco smoke, which has been shown to induce cytotoxic effects in fibroblast cells with IC_50_ values ranging from 6 µM to 25 mM (Holliday et al., 2019). Taken together, these findings suggest that both Cd and nicotine could synergistically contribute to cellular dysfunction and damage in the oral cavity.

Salivary Cd concentrations are significantly elevated in waterpipe and long-term smokers, with levels reported up to 352.72 μM in certain studies.

Understanding these mechanistic effects is critical because they can influence disease progression. Our study showed that Cd increased phosphorylation of Akt, ERK1/2, and JNK, with specific inhibitors blocking these pathways. However, we did not observe significant phosphorylation of p38. This finding contrasts with previous studies showing p38 activation in other cell types such as breast cancer, osteosarcoma, and bronchial epithelial cells, following Cd exposure (Casano et al., 2010; Hu et al., 2015; Cao et al., 2021). This discrepancy may reflect the cell-type-specific nature of p38 MAPK, which is more commonly activated by oxidative stress in immune or neuronal cells (Galán et al., 2000; Rockwell et al., 2004). Additionally, higher Cd concentrations (15–30 μM) were required for p38 activation in mouse hippocampal cells (HT4) (Wang et al., 2019), whereas we limited our analysis to 1 μM Cd, which was the highest concentration that did not induce cell death in our experiment to avoid cytotoxicity. At this lower concentration, Cd appears to preferentially activate Akt, ERK1/2 and JNK, which are more directly linked to inflammatory signaling in our study. This supports the notion that p38 activation is concentration-dependent and more relevant at higher Cd levels. Our findings suggest that Cd-induced pro-inflammatory responses in HGFs is primarily driven by phosphorylation of Akt, ERK1/2, and JNK, with p38 playing a minor role in this context.

We also study how Cd induces pro-inflammatory responses in HGFs by measuring IL-6 and IL-8 levels. These two cytokines play important roles in chronic inflammation and are implicated in periodontal diseases and oral cancers. Cd exposure significantly elevated the secretion of pro-inflammatory cytokines IL-6 and IL-8 in our HGFs. The results are consistent with previous studies demonstrating Cd-induced cytokine upregulation in various cell types, including human astrocytes and breast cancer cells, highlighting its role in promoting pro-inflammatory responses (Phuagkhaopong et al., 2017; Bimonte et al., 2024). Cd also induced epidermal growth factor receptor (EGFR), one of the important factors for IL-1 and IL-6, in breast cancer cells and human lung adenocarcinoma cell lines (A549) (Wei et al., 2015; Kundu et al., 2011). The inflammatory effects of Cd become more concerning when considering environmental sources. As a component of PM2.5, Cd links air pollution to oral inflammation. Exposure to PM2.5 has been associated with higher salivary IL-6 levels in humans (He et al., 2022), which aligns with our findings of Cd-elevated IL-6 in HGFs. Since IL-6 plays a key role in inflammatory responses, tissue inflammation and structural damage in oral tissues after prolonged exposure to PM2.5 were observed in the previous study (Zhu et al., 2024). Additionally, the attenuation of IL-6 and IL-8 secretion by inhibitors targeting the Akt, ERK1/2, and JNK pathways in our study indicates that these pathways mediate the effects of Cd-induced IL-6 and IL-8 cytokine release in HGF cells.

In addition to cytokine release, Cd exposure also increased COX-2 expression, a key mediator of inflammation. In this study, we measured COX-2 expression after a 1-h incubation of CdCl2 in HGFS based on its short half-life (< 3.5 h) and previously observed an increase in COX-2 mRNA within 1-2 hours after tissue injury (Khan et al., 2007; Lukiw et al., 1997). Clinical and animal studies have shown that the peak of COX-2 expression was early at 1-2 h after carrageenin induction (Gilroy et al., 1999). Based on our results, CdCl_2_ did not induce p38 MAPK activation, we focused on Akt, ERK1/2, and JNK which are more relevant to Cd-induced pro-inflammatory responses in HGFs. In our study, Cd-induced COX-2 overexpression was reduced by inhibitors for Akt, ERK, and JNK pathways. Our findings align with previous studies showing Cd-induced COX-2 upregulation through Akt and ERK signaling in various cell types, including RAW264.7 cells and human gallbladder epithelial cells (Huang et al., 2014; Sharma et al., 2020). In animal study, the study in mice showed that Cd administration (5 mg/kg body weight) over 15 days elevated COX-2 and IL-6 levels in lung tissues (Kundu et al., 2009). In contrast, studies in HT4 cells at 15 µM Cd demonstrated that higher Cd concentrations induce COX-2 via the p38 pathway but not JNK (Rockwell et al., 2004). This supports the idea that higher Cd concentrations primarily activate the p38 MAPK pathway, while lower concentrations may preferentially activate the JNK pathway which was observed in our study. These findings emphasize the complexity of Cd-induced signaling pathways and Akt, ERK1/2, and JNK as primary drivers of pro-inflammatory responses in oral fibroblasts, while p38 plays a more prominent role under higher Cd-induced oxidative stress and cytotoxicity. Taken together, our findings highlight that Cd-induced IL-6, IL-8, and COX-2 expression in HGFs is not a non-specific stress effect but is regulated through Akt, ERK1/2, and JNK activation.

While previous studies have explored Cd’s cytotoxic effects (Issa et al., 2008), its correlation with periodontal (Yang et al., 2025), or signaling responses in other tissues (Chmielowska-Bąk and Deckert, 2012; Chen et al., 2022), these aspects were often examined separately. In contrast, our study combines cell viability, cytokine secretion, and COX-2 expression with mechanistic validation using pathway-specific inhibitors. This provides a mechanistic basis for the pro-inflammatory response observed in Cd-exposed oral tissues and distinguishes our study from prior works that did not examine intracellular signaling cascades in gingival fibroblasts. Moreover, we used Cd concentrations that reflect environmental or secondhand smoke exposure which differs from the higher levels used in earlier studies. This makes our findings more applicable to real-world oral health scenarios.

However, the present study has certain limitations. Inflammation is a complex biological process involving multiple cell types, vascular components, and molecular mediators, whereas our single-cell *in vitro* model focuses on intrinsic cellular immune responses, such as cytokine production and intracellular signalling. As a result, our model captures only intrinsic cell-autonomous responses and cannot fully replicate the interactions among different cell types that occur during inflammation. Future studies incorporating other cell types would further enhance the physiological relevance of the findings. Additionally, as this study used HGFs from a single donor, and genetic variability influences susceptibility to periodontitis, the response to Cd may differ across individuals. Including HGFs from diverse donors and other oral cell types in future work would help address this limitation. Lastly, *in vivo* studies are essential to validate our results and assess the long-term effects of Cd exposure on oral health.

In conclusion, this study highlights Cd's harmful effects on HGFs through activation of the Akt and MAPK (ERK1/2, JNK) signaling pathways. This leads to increased IL-6, IL-8, and COX-2 expression, contributing to oral pro-inflammatory responses. The findings support future research on targeting these pathways to reduce Cd-induced inflammation and the public health impact of smoking and environmental Cd exposure.

## Data Availability

The original contributions presented in the study are included in the article/Supplementary Material, further inquiries can be directed to the corresponding author.
