# Supplementary figures and images for: Cadmium decreases human gingival fibroblast viability and induces pro-inflammatory response associated with Akt and MAPK pathway activation
Source: Front Toxicol. 2025 Jul 23;7:1583865. doi: 10.3389/ftox.2025.1583865 (PMC12325209; doi:10.3389/ftox.2025.1583865)

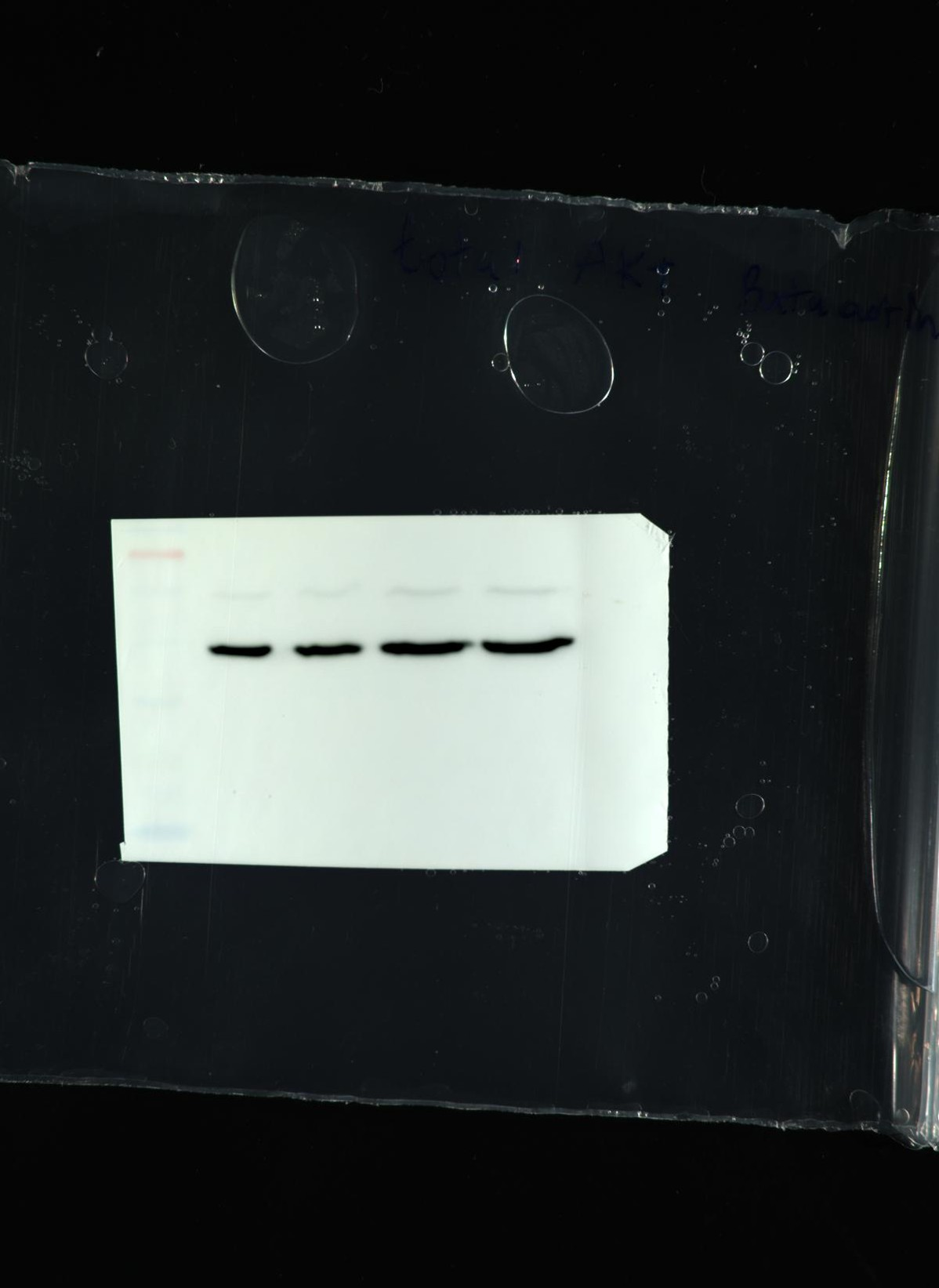

Supplement: Supplementary file 1 [file Image3.TIFF]

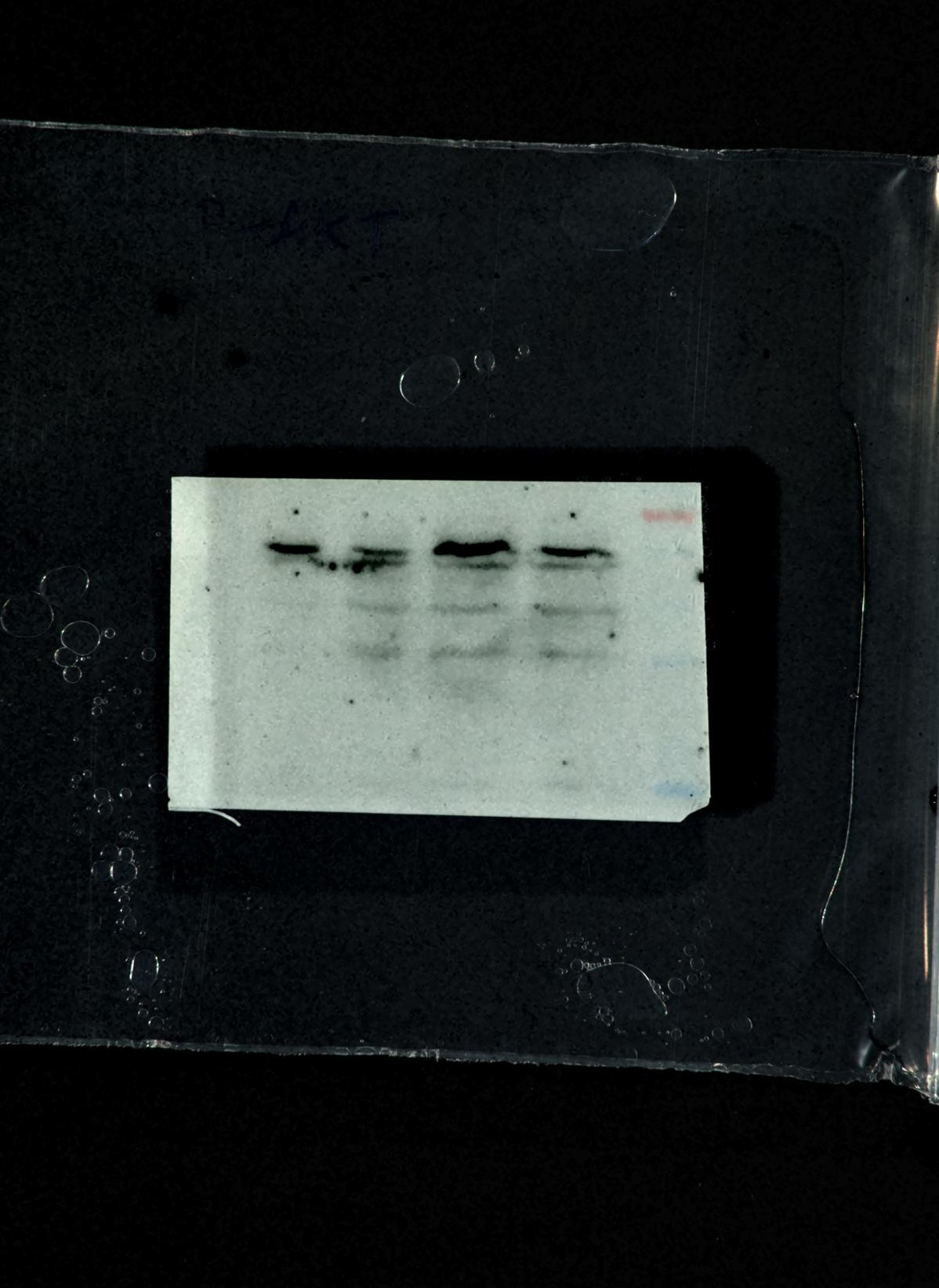

Supplement: Supplementary file 2 [file Image1.TIFF]

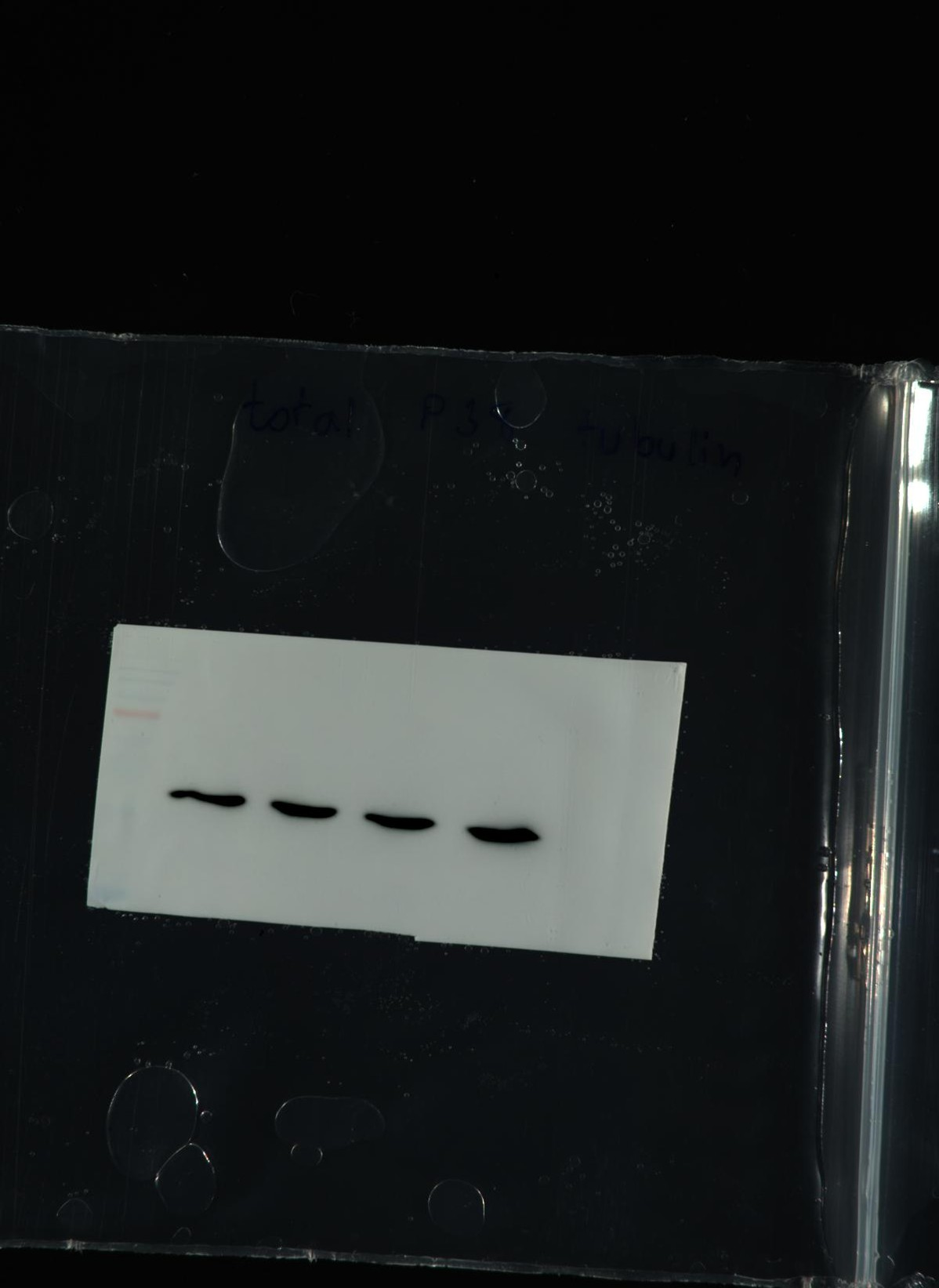

Supplement: Supplementary file 3 [file Image9.TIFF]

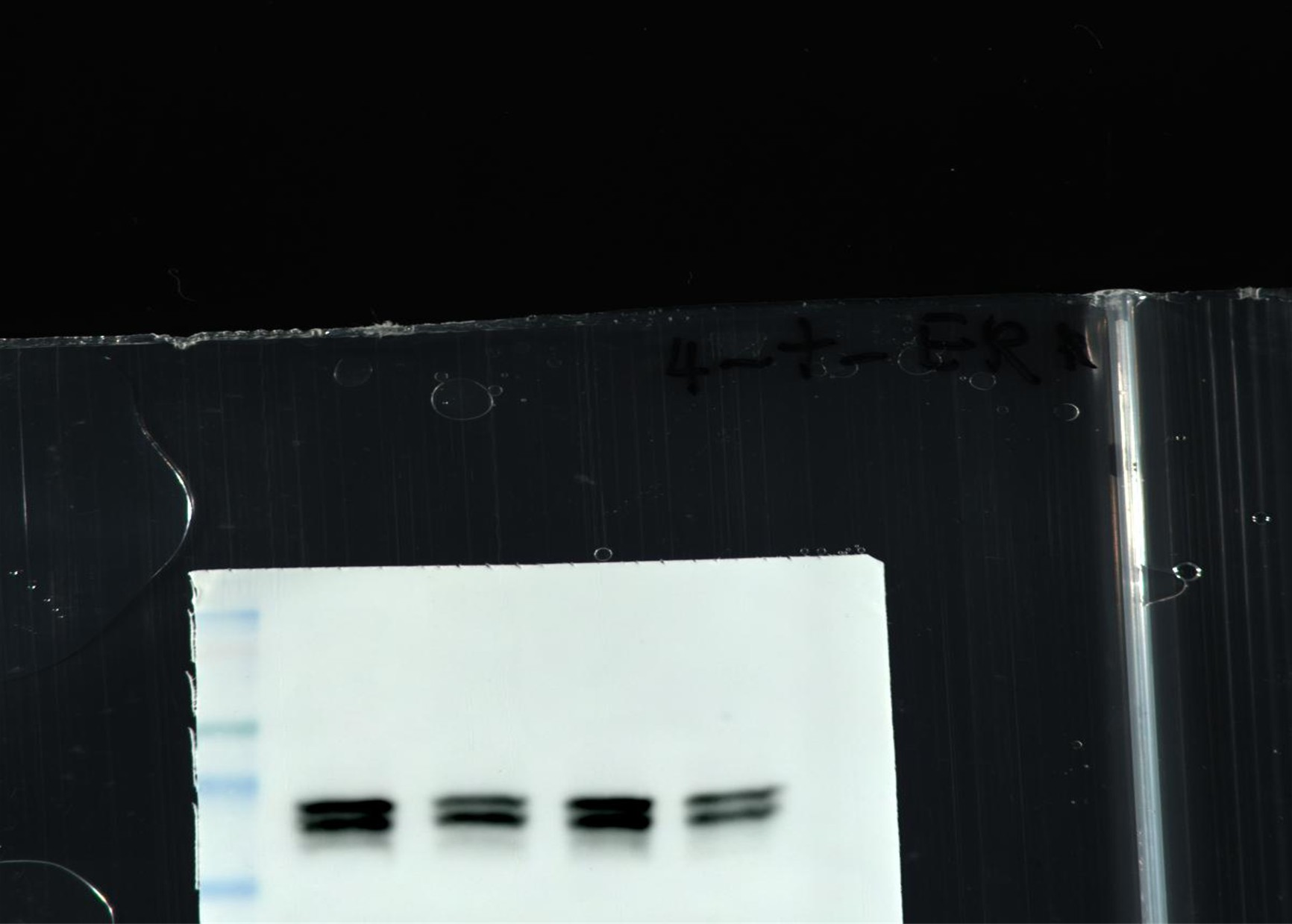

Supplement: Supplementary file 4 [file Image5.TIFF]

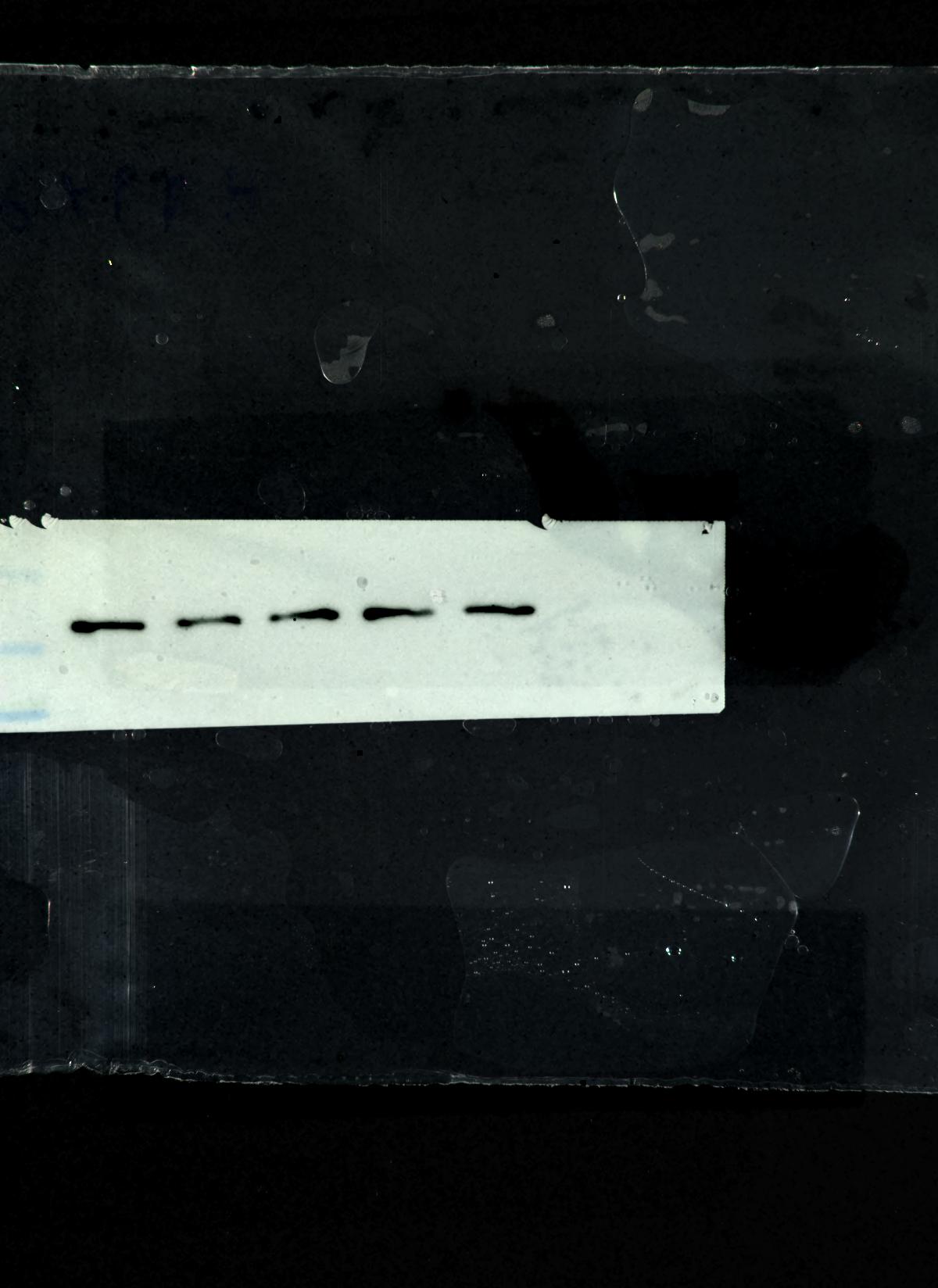

Supplement: Supplementary file 5 [file Image14.JPEG]

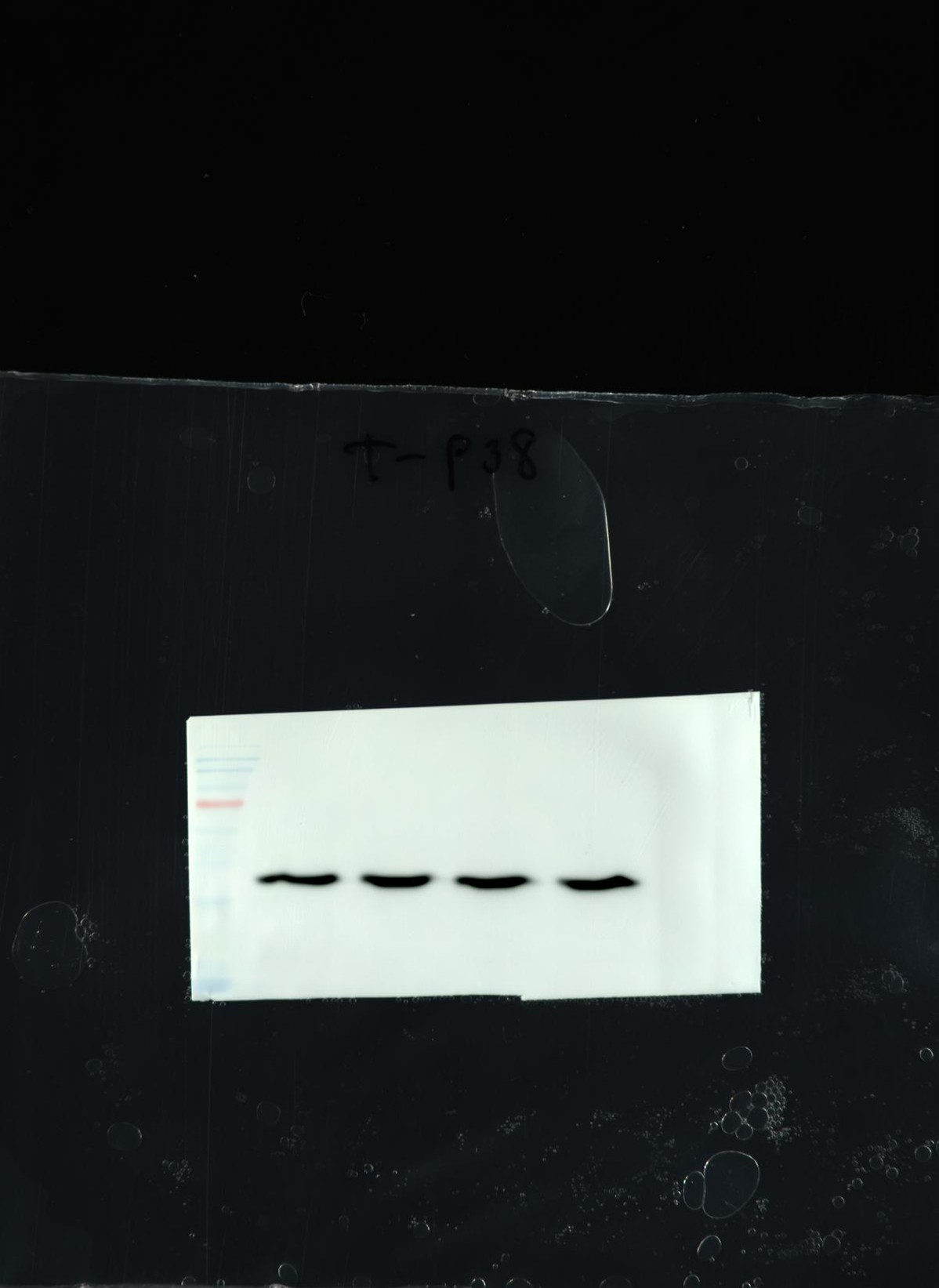

Supplement: Supplementary file 6 [file Image8.TIFF]

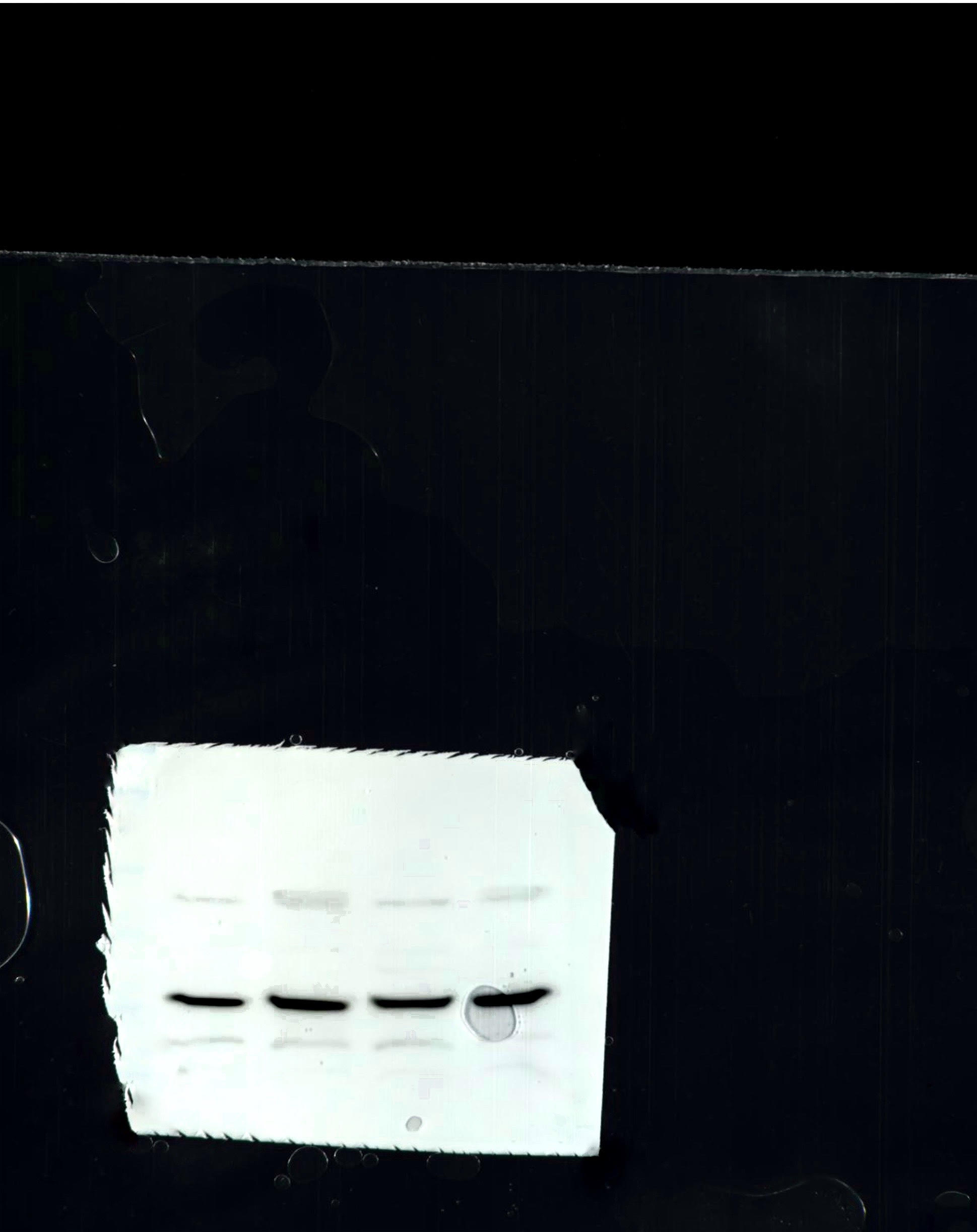

Supplement: Supplementary file 7 [file Image11.TIFF]

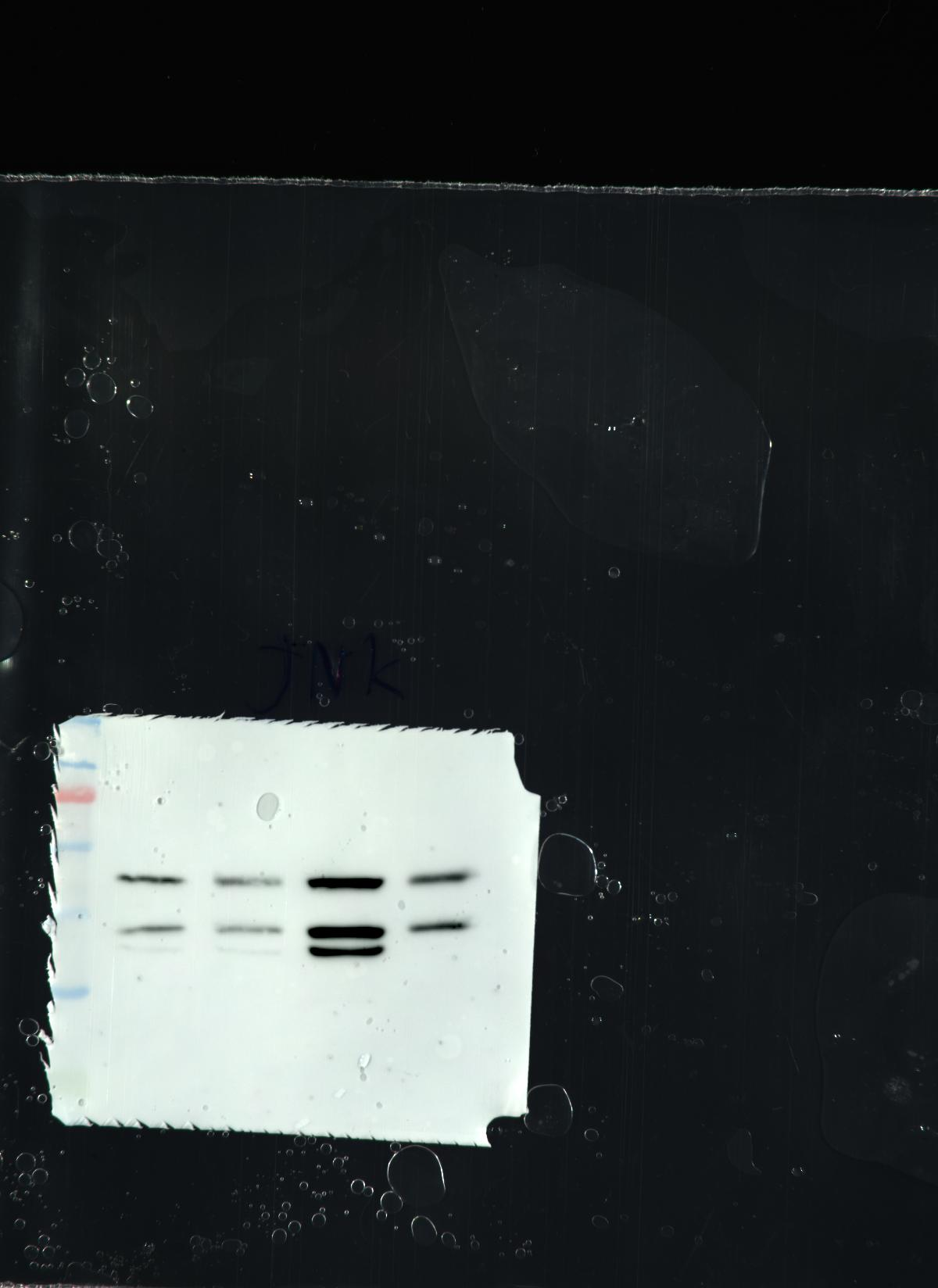

Supplement: Supplementary file 8 [file Image10.TIFF]

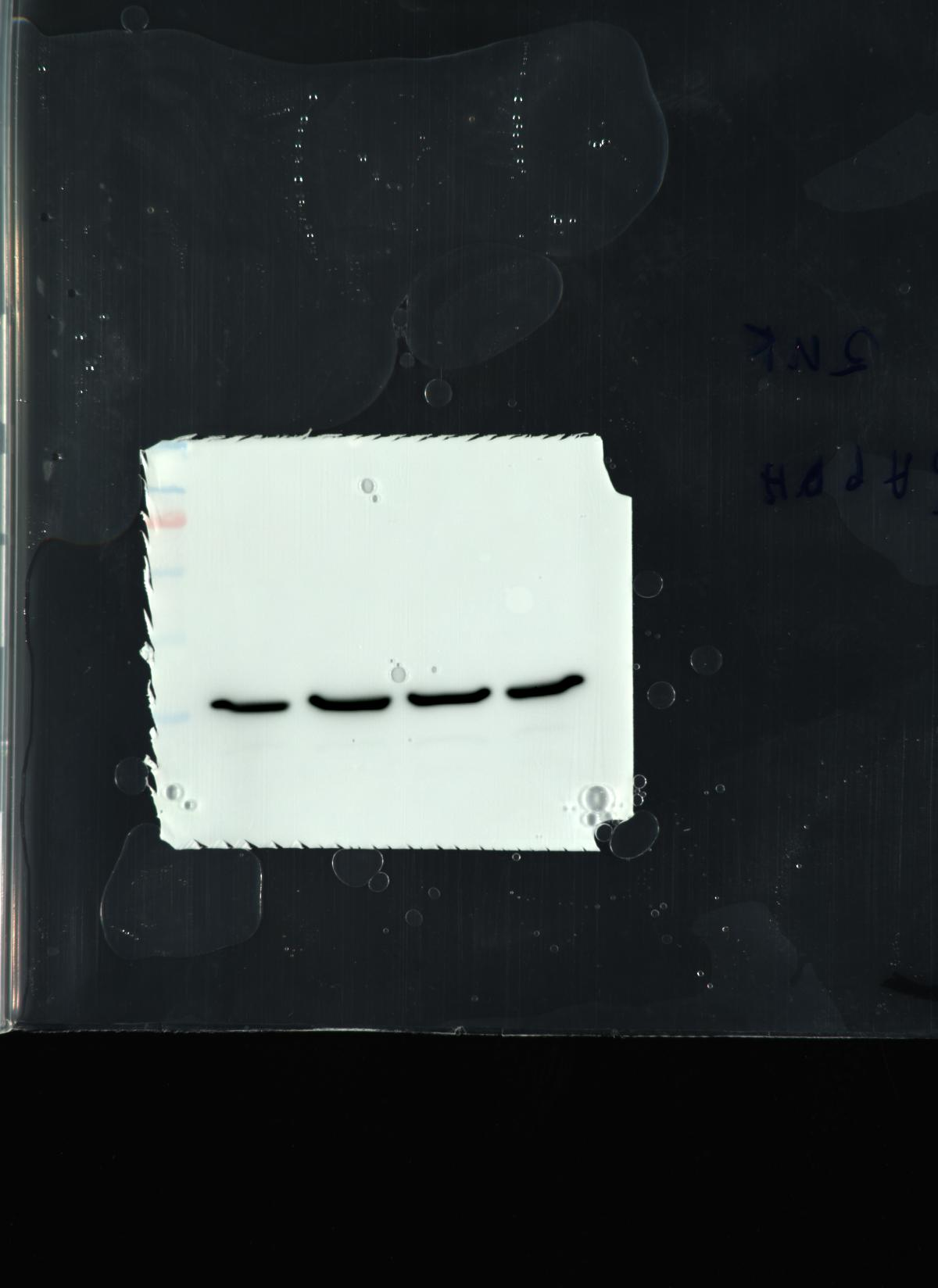

Supplement: Supplementary file 9 [file Image12.TIFF]

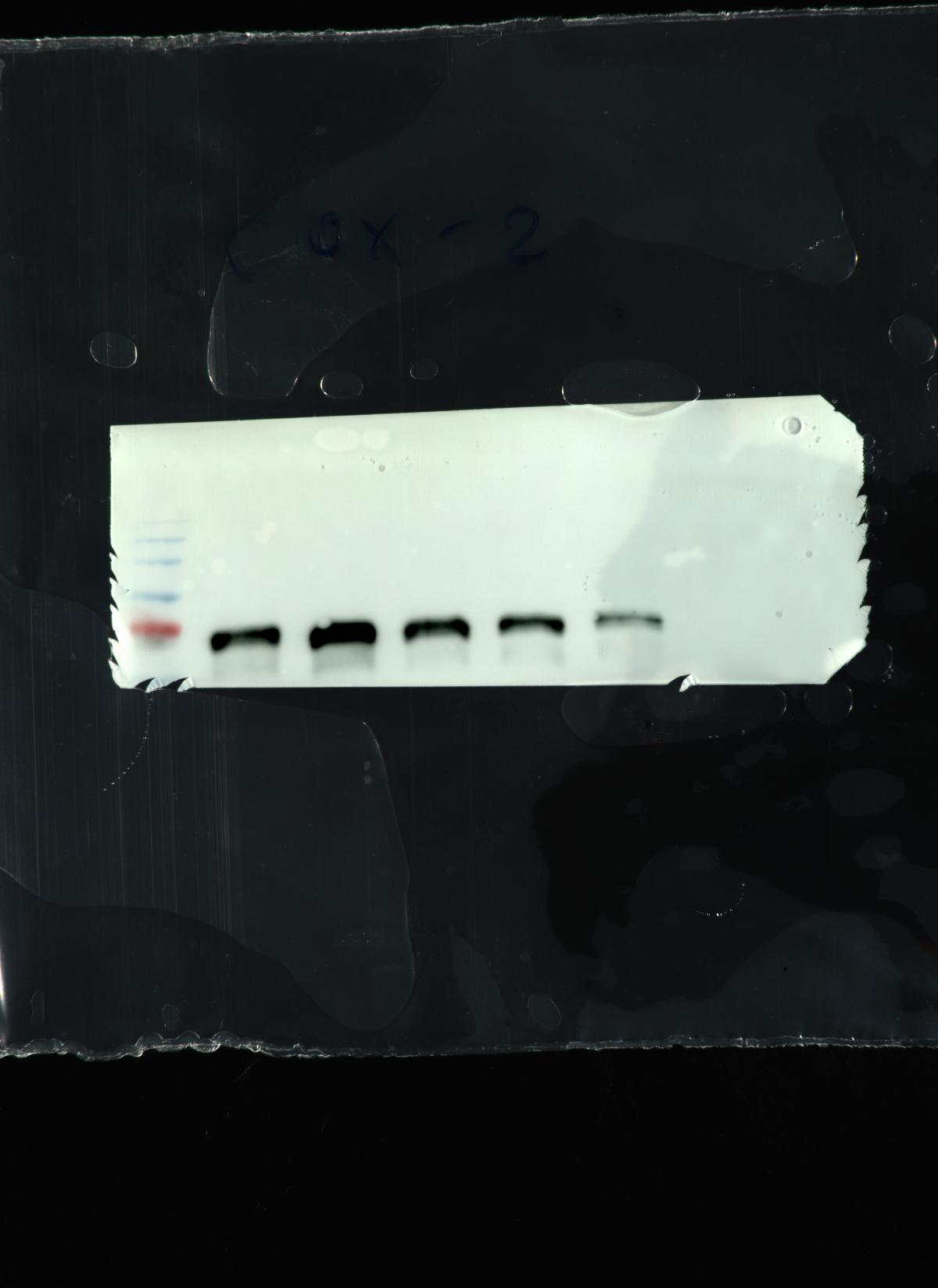

Supplement: Supplementary file 10 [file Image13.JPEG]

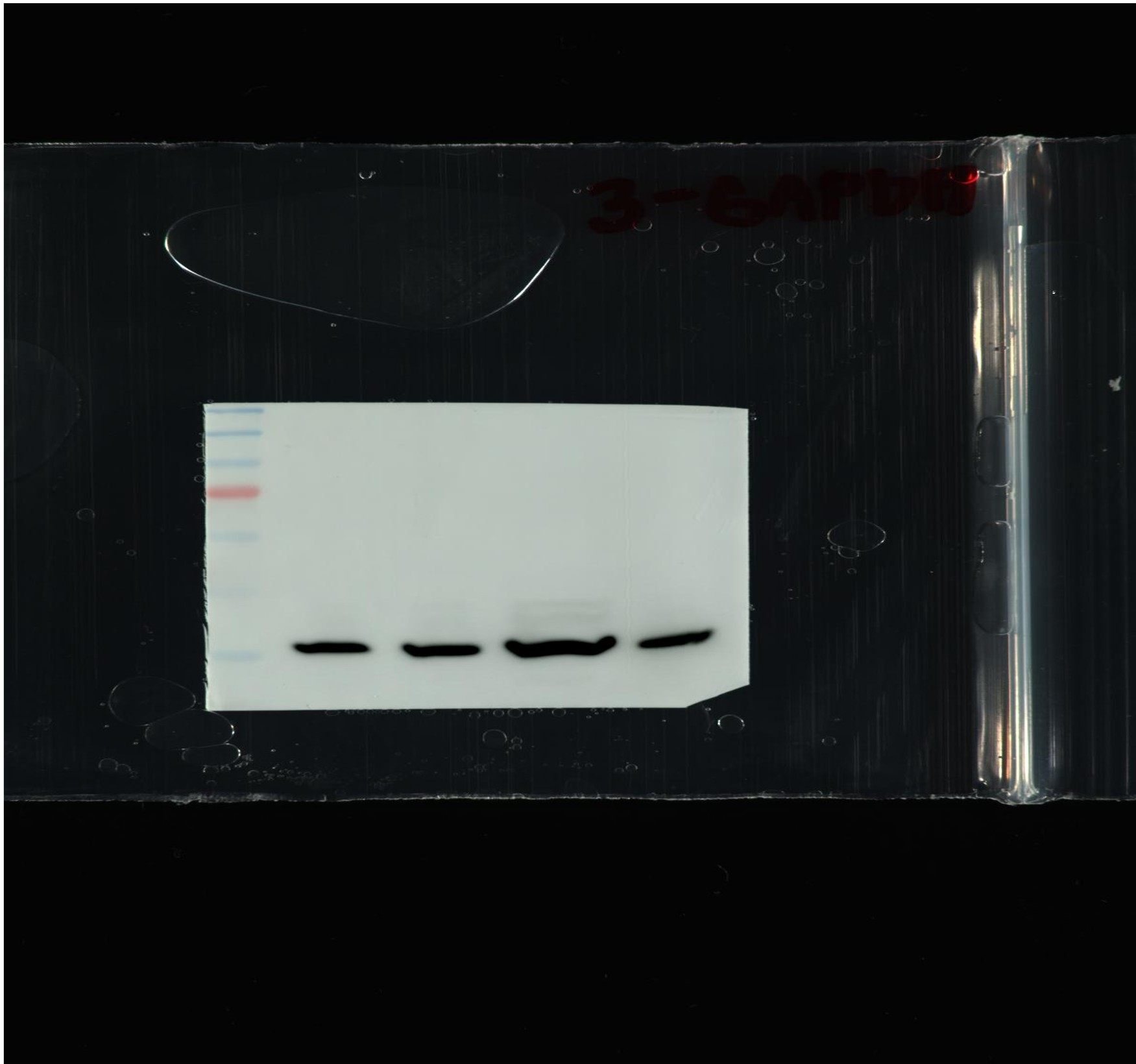

Supplement: Supplementary file 11 [file Image6.TIFF]

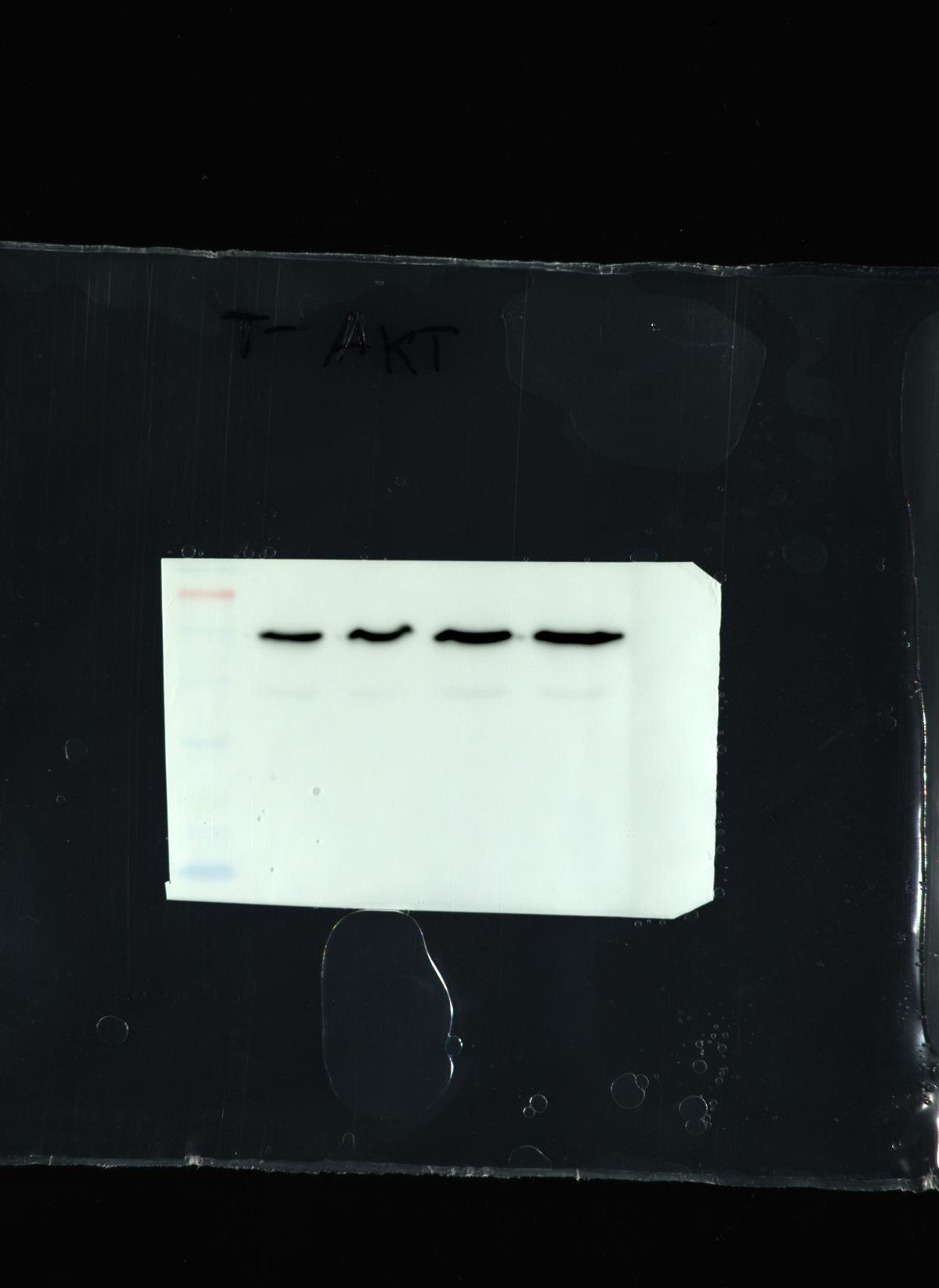

Supplement: Supplementary file 12 [file Image2.TIFF]

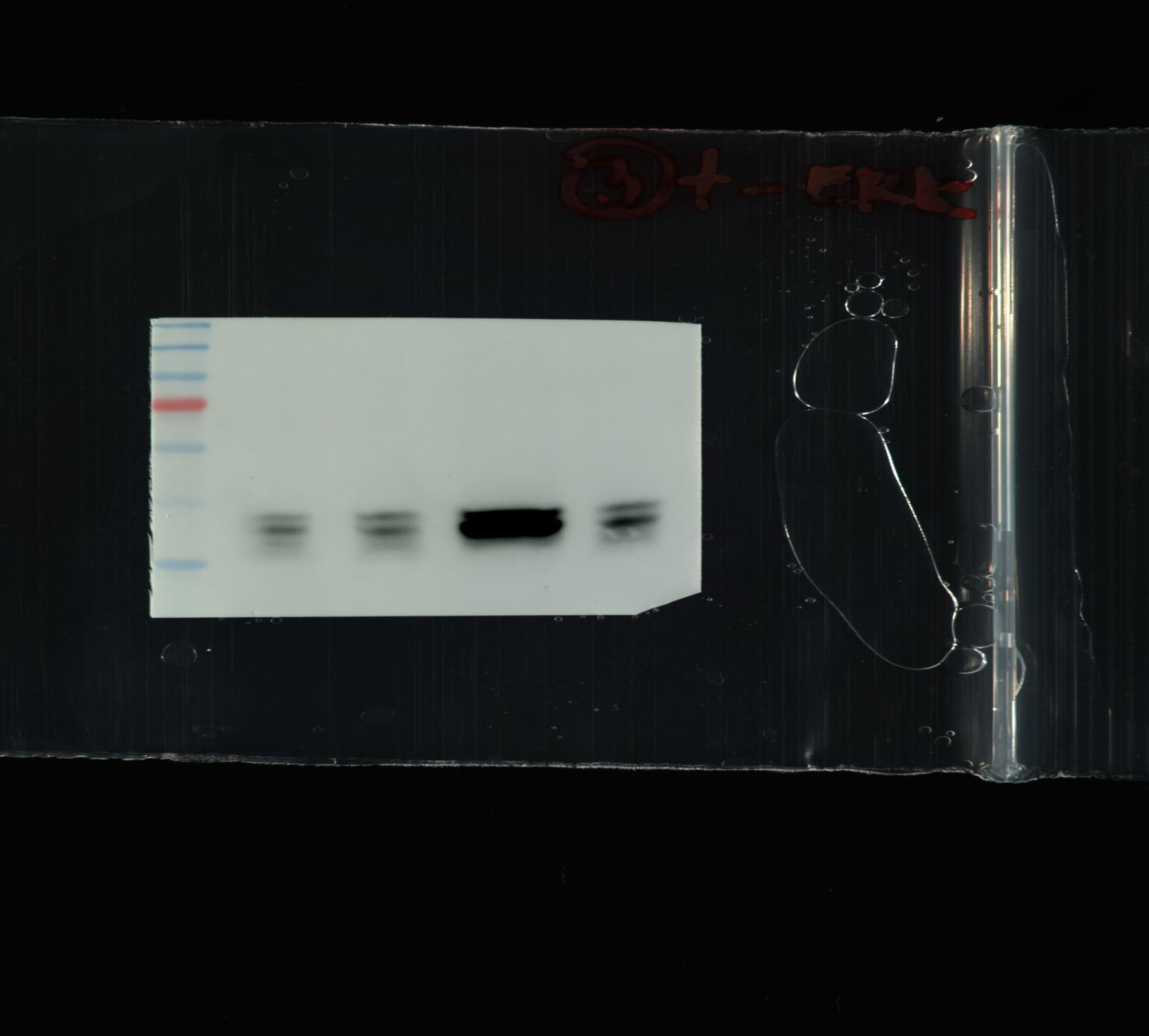

Supplement: Supplementary file 13 [file Image4.TIFF]

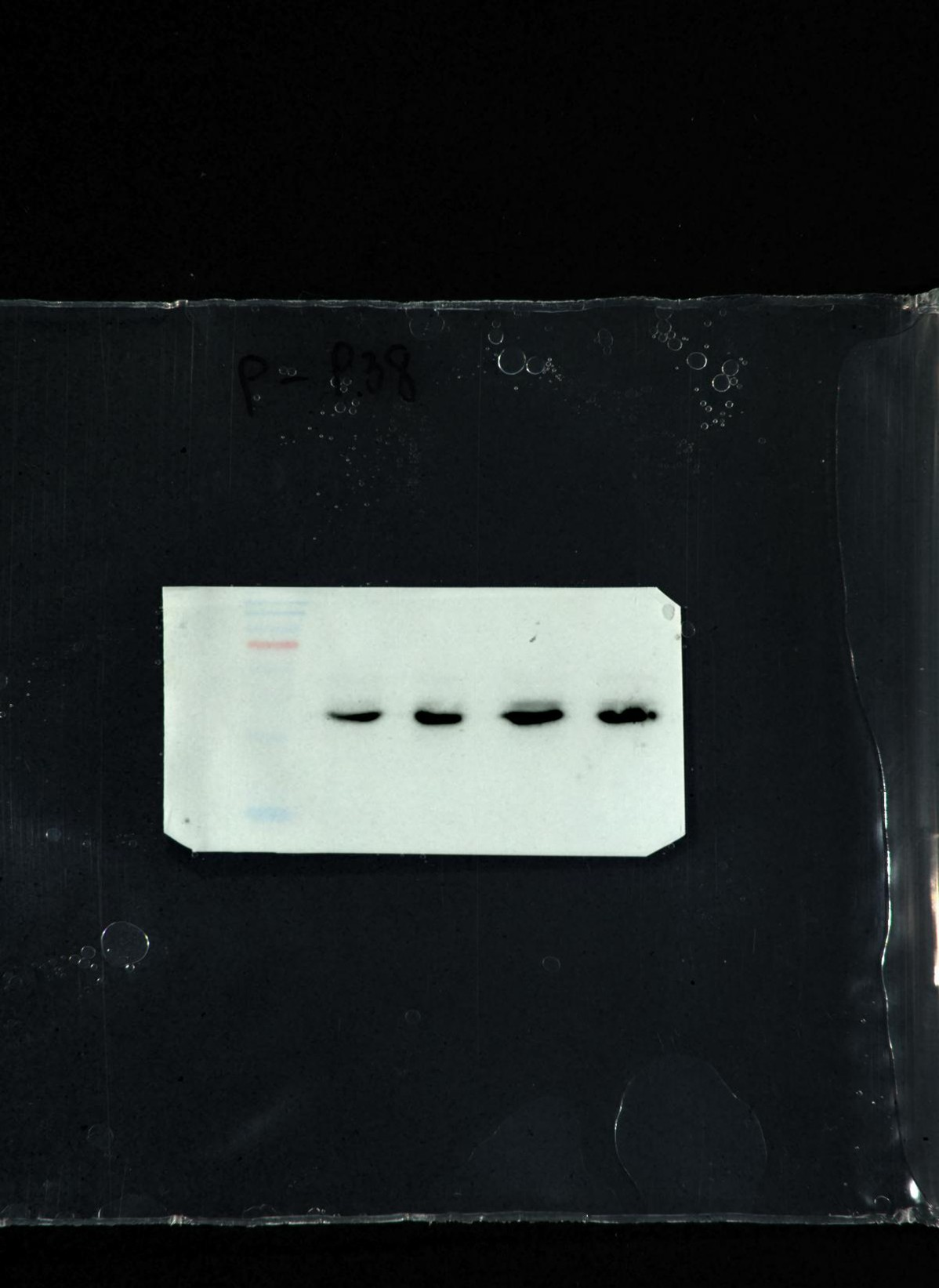

Supplement: Supplementary file 14 [file Image7.TIFF]
